# Solitary plasmacytoma of the sternum with a spiculated periosteal reaction: A case report

**DOI:** 10.3892/ol.2014.2636

**Published:** 2014-10-24

**Authors:** JINGPIN ZHAO, YUQING LI, WENJUAN WU, ZEKUN ZHANG, YANG DING

**Affiliations:** Department of Radiology, Third Hospital of Hebei Medical University, Hebei Province Biomechanical Key Laboratory of Orthopedics, Shijiazhuang, Hebei 050051, P.R. China

**Keywords:** plasmacytoma, sternum, spiculated periosteal reaction, sunray

## Abstract

Solitary plasmacytomas (SPs) represent ≤5% of all plasma cell neoplasms and mostly occur in the spine, pelvis, ribs and pectoral girdle, while rarely occurring in the sternum. The tumors typically appear as osteolytic lesions. In rare cases, SPs can manifest as bony spicules on the surface of the bone. The present study reports the case of a 74 year-old female with an osteolytic tumor localized in the sternum. The tumor displayed extensive bony destruction, with a large quantity of thick straight spicules on the surface of the bone, resembling a sunray in appearance. The imaging, laboratory and pathological examinations of the patient met the diagnostic criteria of SP. The patient was initially treated with radiotherapy at a dose of 45 Gy. Six months later, chemotherapy consisting of vindesine, Adriamycin and dexamethasone was administered. Vindesine and Adriamycin were administered at a dose of 2 and 15 mg/day, respectively on days 1–4 in a 20-day cycle. Dexamethasone was administered at a dose of 20 mg/day on days 1–4, 9–12 and 17–20 in the 20-day cycle. In total, the patient underwent 6 cycles of chemotherapy, with a total duration of 7 months. The patient was followed-up for two years after beginning therapy. At present, the patient is well, without any evidence of progressive disease or multiple myeloma. To the best of our knowledge, this is the first case in the English literature of SP in the sternum, with an unusual sunray periosteal reaction on radiological imaging. The sites of bony spiculation in the lesions that have previously been described in the literature are the mandible, orbit, vertebral body and skull vault. To the best of our knowledge, the current study presents the first case of a SP of the sternum with a unusual spiculated periosteal reaction on radiological imaging to be reported in the English literature.

## Introduction

Solitary plasmacytomas (SPs) are rare and are characterized by a localized accumulation of neoplastic monoclonal plasma cells, without proof of systemic myelomatosis. The tumors represent ≤5% of all plasma cell neoplasms (1) and mostly occur in the spine, pelvis, ribs and pectoral girdle (1–3), while rarely occurring in the sternum. Radiologically, SP of the bone typically appears as an osteolytic lesion; the tumor usually destroys the cortex in several places and invades the soft tissues (2,4). In rare cases, the radiological findings may take unusual forms, for example, they can manifest as bony spicules on the surface of the bone, which resemble the appearance of sunrays (5–12). The sites of bony spiculation in the lesions previously described in the literature are the mandible, orbit, vertebral body and skull vault (5–12).

The current study presents a case of a solitary plasmacytoma of the sternum with a spiculated periosteal reaction on radiological imaging. Written informed consent was obtained from the patient.

## Case report

A 74-year-old female presented with neck and anterior chest wall pain, along with swelling in the chest area that had persisted for one month. The past medical history consisted of type II diabetes mellitus and hypertension. There was no history of cancer. A physical examination revealed an ill-defined swelling on the anterior chest wall. The area was tender and no pulsation was noted. Routine urinalysis and hematological and biochemical tests were within the normal limits. A urine test for Bencae Jones protein was negative.

**Figure 1:**
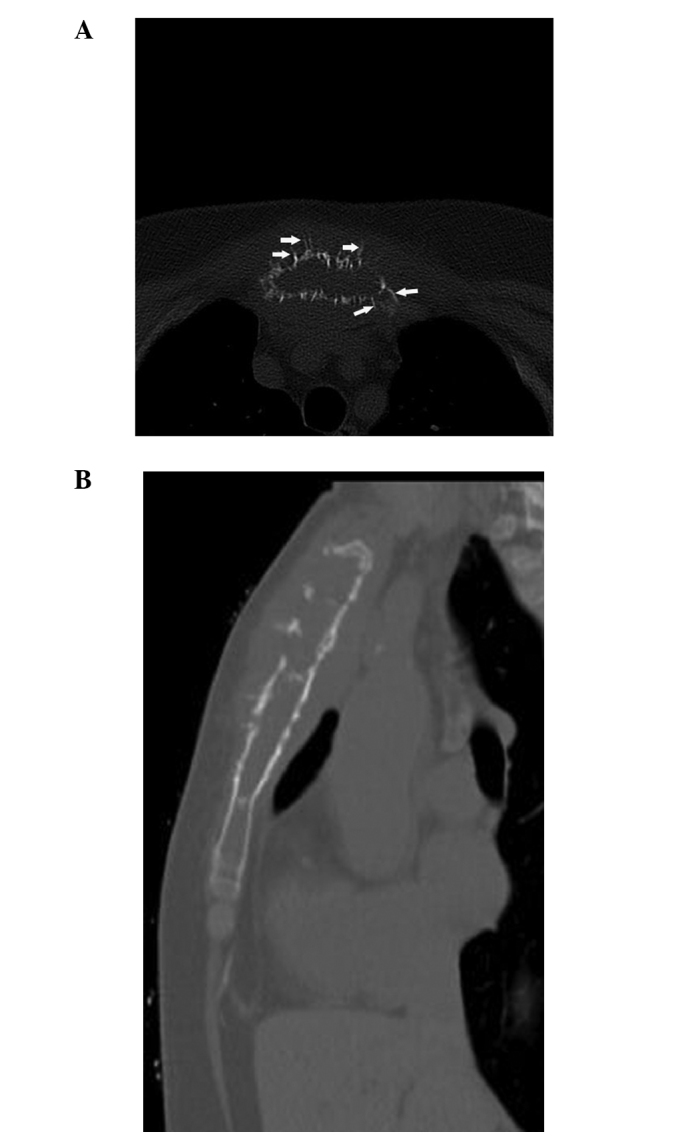
(A) Axial computed tomography (CT) image of the manubrium sterni obtained through the bone window showing an osteolytic lesion with expansion and a periosteal reaction with the appearance of sunrays around the periphery (arrows). (B) Sagittal reconstructed CT image of the sternum showing the lesion involving the manubrium and almost all the body of the sternum. The lesion is slightly expansile and the cortex is partially destroyed.

**Figure 2:**
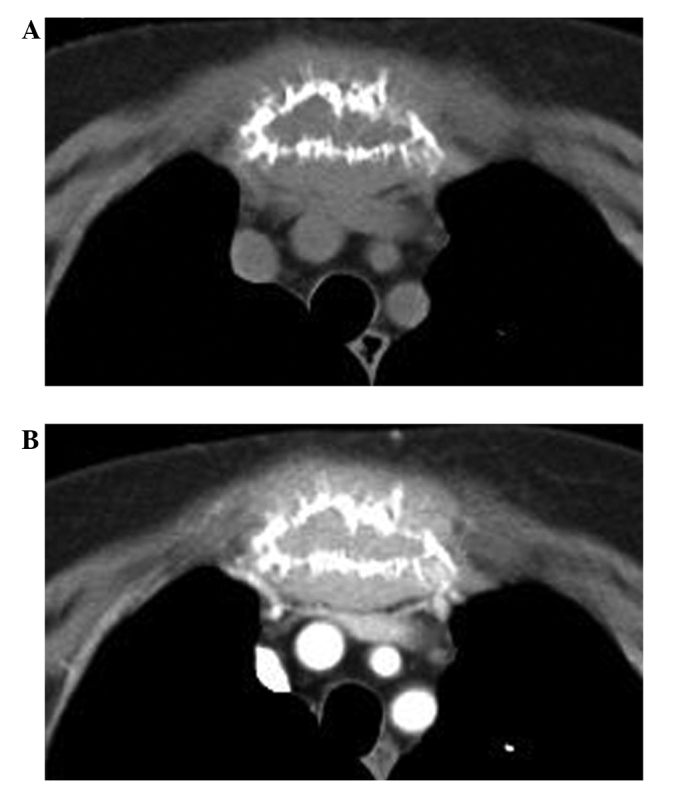
(A) Axial computed tomography (CT) image of the manubrium sterni obtained through the soft tissue window showing that the bone marrow of the sternum has been substituted by homogeneous soft tissue. (B) Axial contrast-enhanced CT image of the manubrium sterni showing marked homogeneous enhancement of the tumor.

**Figure 3:**
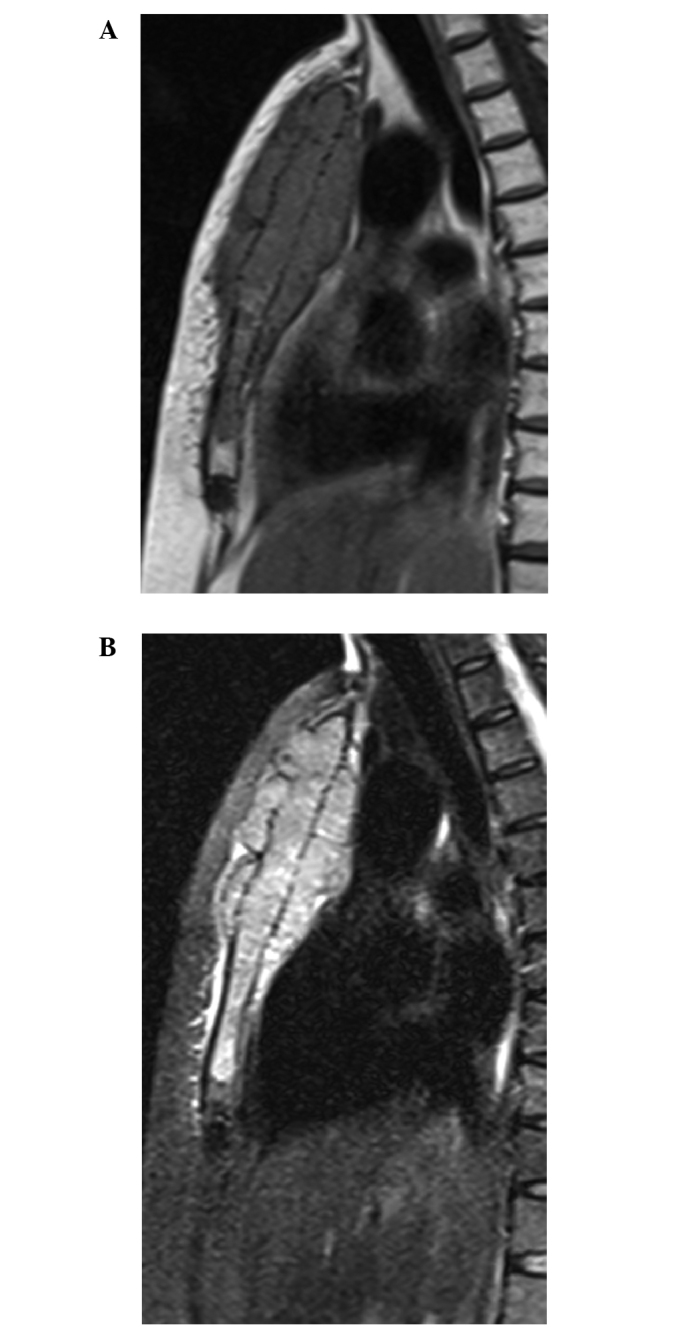
(A) T1-weighted sagital image (repetition time/echo time, 590/21 msec) of the sternum showing an area of low signal intensity in the manubrium and body of the sternum. (B) Short-τ inversion recovery T2-weighted sagittal image (repetition time/echo time, 4150/106 msec) of the sternum showing an area of high intensity in the same region; the soft-tissue mass is clearly demonstrated.

Conventional radiography of the chest was normal. On the axial and sagittal computed tomography (CT) scans, the manubrium and body of the sternum had extensive bony destruction, with a large quantity of thick straight spicules on the surface of the bone resembling a sunray in appearance. The lesion had slight expansion and disruption of the cortex in certain places and had spread to the adjacent soft tissues. There was no calcification in the lesion (Fig. 1). The tumor exhibited marked homogeneous enhancement on contrast-enhanced CT (Fig. 2). Magnetic resonance (MR) imaging showed that the lesion displayed a homogeneous low signal intensity on T1-weighted images, and a high signal intensity on short-τ inversion recovery T2-weighted images. The soft-tissue mass was clearly demonstrated (Fig. 3). Use of ^99m^Tc-labelled whole-body bone scintigraphy disclosed normal results, with the exception of increased uptake in the sternum.

**Figure 4:**
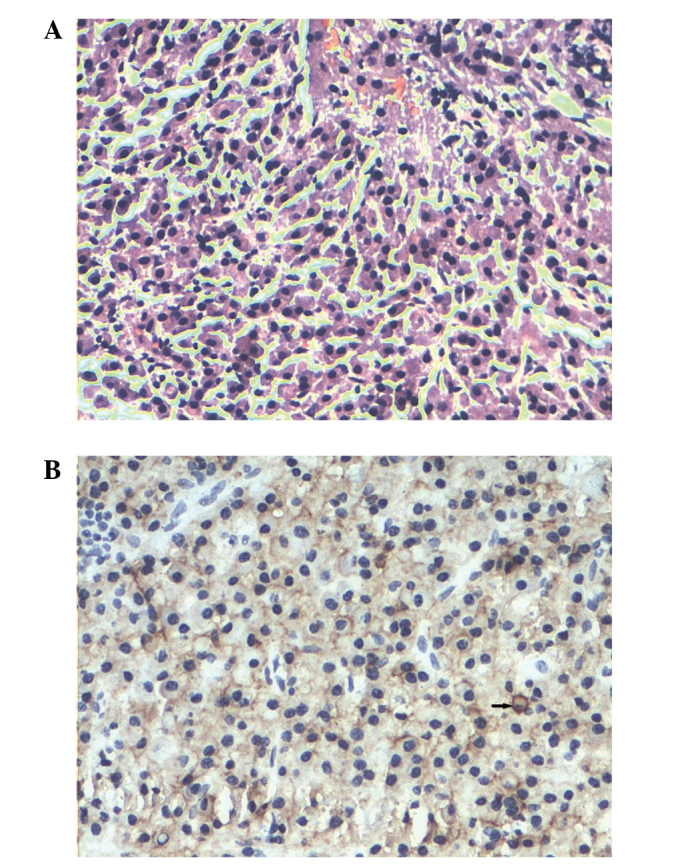
(A) Histopathological examination (hematoxylin and eosin stain; original magnification, ×200) demonstrating proliferation of round cells with abundant cytoplasm and eccentric nuclei with coarse chromatin, indicating a plasmacytoma. (B) Immunohistochemical analysis revealing positive cluster of differentiation 38 staining on the cell membrane (arrow), which is characteristic of a plasmacytoma (original magnification, ×200).

In order to obtain the correct diagnosis, an open biopsy was performed at the sternum. The histopathological examination demonstrated that the destroyed bone marrow had been substituted with abundant neoplastic plasma cells with eccentric ‘clock-face’ nuclei and copious cytoplasm. Immunohistochemical staining was positive for immunoglobulin (Ig) λ- and κ-light chain antibodies, and cluster of differentiation (CD)38 and -138 (Fig. 4). The histological diagnosis of the tumor was of a plasmacytoma. Immunoelectrophoresis of the serum proteins revealed mild monoclonal gammopathy of IgA (IgA λ-type). Iliac bone marrow aspiration revealed no evidence of myeloma (<5% plasma cells). MR imaging of the cervical and lumbar spine, a CT scan of the head and thorax, and a radiograph of the pelvis revealed no other skeletal or extraskeletal lesions. The overall findings met the diagnostic criteria of SP, therefore, multiple myeloma was ruled out and the diagnosis of SP was established.

The patient was initially treated with radiotherapy at a dose of 45 Gy delivered in 15 fractions over 21 days. Half a year later, chemotherapy consisting of vindesine (2 mg/day, days 1–4), Adriamycin (15 mg/day, days 1–4) and dexamethasone (20 mg/day, days 1–4, 9–12 and 17–20) was administered. In total, the patient underwent 6 cycles of chemotherapy, with a total duration of 7 months. The patient was followed for two years after beginning therapy, without any signs of progressive disease or conversion to multiple myeloma.

## Discussion

The association between SP and multiple myeloma is currently unclear. Certain individuals regard SP as a distinct entity from multiple myeloma, while others regard SP as an early presentation of multiple myeloma (3). The diagnostic criteria of SP of the bone requires the presence of a solitary bone lesion confirmed by skeletal survey, plasma cell infiltration proven by biopsy, the absence of myeloma cells in the bone marrow and a lack of anemia, hypercalcemia and renal involvement (13). The average age of onset of SP is approximately one decade less than that for multiple meyloma, with a median age of 56 years old (3). The male to female ratio is approximately 2:1 (1). SP of the sternum is rare. The majority of neoplasms of the sternum are metastases. Primary sternal neoplasms are uncommon and are much more frequently malignant than benign (2). In a study of the Chinese literature that reported 30 cases and reviewed a total of 248 cases of sternal tumors, the most malignant tumors were metastases, followed by myelomas, chondrosarcomas, lymphomas and osteosarcomas; the most benign tumors were chondromas and osteochondromas (14).

Sternal plasmacytoma usually presents as an osteolytic expansile lesion or a typical ‘punched-out’ lytic lesion on radiological examination (15). The tumor usually destroys the cortex in several places and invades the soft tissues (2,4). CT may demonstrate a subtle lytic lesion or small soft-tissue masses of the sternal lesion that are not visible by radiography (16). On MR imaging, the tumors usually exhibit a low signal intensity on T1-weighted images and a high signal intensity on T2-weighted images (2).

In the present case, the tumor appeared as an extensive, mildly expansile lytic lesion, with destruction of the cortex and adjacent soft tissue mass. Around the periphery of the lesion, there were straight bony spicules extending outward from the cortex that resembled sunrays in appearance. To the best of our knowledge, there have only been a few previous case studies with bony spicules forming the appearance of sunrays (5–12). The majority of these cases occurred in the mandible, while other involved sites consisted of the orbit, vertebral body and skull vault (5–12). The majority of these cases were multiple myeloma; only two of the cases were SP (6,11). Mohammadi *et al* reported a case of solitary mandibular plasmacytoma, which displayed a lytic lesion with a sunray periosteal reaction on the conventional radiograph (6). Lipper *et al* reported a solitary osteosclerotic mandibular plasmacytoma with prominent sunray spiculation, which radiologically mimicked an osteosarcoma (11). This unique appearance can be explained by the fact that any malignant cell has the potential ability to stimulate the formation of new bone (6). Considering the radiological appearance of the present case, malignant tumors should be considered. Metastatic disease is the most common differential diagnosis; the metastases are often multiple in number, with a larger soft-tissue mass. In the present case, negative bone scintigraphy everywhere except the sternum indicated a low possibility of metastatic disease. The most common primary malignant tumor is chondrosarcoma. It usually occurs in patients in the fourth to sixth decades of life, and frequently appears as a large, lobulated mass with multiple stippled calcifications (15). In the present case, there were no calcifications in the lesion. Other conditions that can manifest with a sunray appearance are osteosarcoma and Ewing’s sarcoma. Osteosarcomas of the sternum generally occur in older patients (median age, 42 years) compared with those of the extremities (15). A mixed pattern consisting of osteolysis and sclerosis is typical. With respect to the present case, an expansile lytic lesion in a 74-year-old female is an uncommon presentation for osteosarcoma. Ewing’s sarcoma is commonly observed in young individuals, with 80% of cases recorded in patients <20 years of age (5); this condition was almost ruled out in the present case. Another relatively common malignant tumor that occurs in the sternum is lymphoma, which usually demonstrates permeated or moth-eaten bony destruction with an extensive apparent mass. Occasionally, a laminated periosteal bone reaction can be observed (5), however, an expansile lytic lesion with a sunray periosteal reaction is rarely observed in lymphoma.

In conclusion, malignant tumors should be considered if an osteolytic lesion with destruction of the cortex and soft tissue mass is detected in the sternum. Although unusual, a diagnosis of plasmacytoma must be considered in the presence of a lytic lesion with peripheral bony spicules of sunray appearance.
